# Catarrhal Pneumonia in Infants at the Breast

**Published:** 1871-07

**Authors:** 


### CATARRHAL PNEUMONIA OF INFANTS AT THE BREAST.

### *Treatment by Carbonate of Ammonia.*

Dr. Steirlin observes that the danger of catarrhal pneumonia (bronchopneumony, lobular pneumonia) in infants, whether consequent onthe measles, whooping-cough, or bronchial catarrh, is due to the circumstance that the bronchiæ become plugged, carbonic acid narcosis succeeds, and too small a quantity of blood enters the left ventricle, which exerts a paralyzing influence upon the heart. Dr. Steirlin formerly treated such little patients with emetics, as ipecacuana and vinum antimoniale, calomel and mustard poultices, but with very indifferent results. But after he had begun to administer ammonium carbonicum (carbonate of ammonia) and ammonium carbonicum pyro-oleosum, he was so successful in the treatment of this disease, which according to Rilliet and Barthez, is absolutely fatal during the first year of life, that of 150 patients only seven died. He usually prescribed the ammonium carbonicum to the extent of 20 grains; or the liquor ammonium carbon, pyro-oleosi to the extent of half a drachm in two ounces of fluid, of which a teaspoonful should be taken every hour. During the first year of life, besides the above, he applied sinapisms to the chest. In older and stronger children he also prescribed emetics. He never observed any disadvantageous effects.—*Practitioner*, Nov., 1870, from *Berliner Wochenschrift.*

